# Characterization of the woody biomass feedstock potential resulting from California’s drought

**DOI:** 10.1038/s41598-020-57904-z

**Published:** 2020-01-23

**Authors:** Carmen L. Tubbesing, José Daniel Lara, John J. Battles, Peter W. Tittmann, Daniel M. Kammen

**Affiliations:** 1 Department of Environmental Science, Policy, and Management, University of California Berkeley, Berkeley, CA, USA; 2 Energy and Resources Group, University of California Berkeley, Berkeley, CA, USA; 3 Renewable and Appropriate Energy Laboratory, University of California Berkeley, Berkeley, CA, USA; 4 Forest Products Laboratory, University of California Berkeley, Berkeley, CA, USA; 5 Goldman School of Public Policy, University of California Berkeley, Berkeley, CA, USA

**Keywords:** Forest ecology, Environmental impact, Climate-change mitigation, Energy policy

## Abstract

Regional tree die-off events generate large quantities of standing dead wood, raising concern over catastrophic wildfire and other hazards. Governmental responses to tree die-off have often focused on incentivizing biomass energy production that utilizes standing dead trees removed for safety concerns. However, the full distribution of potential woody bioenergy feedstock after tree die-off has not been evaluated due to the complexities of surveying and precisely measuring large forested areas. In this paper, we present a novel method for estimating standing dead biomass at a fine spatial resolution that combines aerial survey data with forest structure maps. Using this method, we quantify biomass generated by the unprecedented tree die-off that occurred in California following a 4-year drought and widespread pest outbreaks. The results are used to estimate feasibly recoverable feedstock for energy production. We find that approximately 95.1 million bone-dry tons (BDT) of dead biomass resulted from 2012–2017 mortality, with a lower bound of 26.2 million BDT. In other words, of the aboveground live tree biomass in 2012, ~1.3–4.8% died by 2017. Of the standing dead biomass, 29% meets minimum constraints for potential cost-effective bioenergy feedstock. This proportion drops to as low as 15% in the most affected areas due to terrain slope, wilderness status, and other factors, highlighting the need to complement disposal via biomass energy with other strategies to mitigate the risks of the tree mortality crisis, which is likely to only become more severe over time due to climate change.

## Introduction

As global mean temperatures rise and regional droughts become more common and severe, epidemics of tree mortality are increasingly threatening the world’s forests^1,2^. In many forests adapted to frequent fires, the risks posed by drought are exacerbated by fire suppression and management legacies. These policies have created more crowded forests where trees compete more strongly for water resources^3,4^. As a result, the forest as a whole is less resilient to drought effects^5,6^. For example, the 2012–2015 drought in California triggered unprecedented tree die-off^5,7^. The United States Forest Services (USFS) estimates that 123 million trees died across California from 2012 to 2017, over five times the mortality detected in the prior six-year period (see Supplementary Table S1)^8^. In some areas, mortality reached over 50% of live tree biomass^9^. Tree mortality crises like the one in California raise critical questions, such as how to address risks associated with their aftermath. Though mortality events run their course over years, the consequences must be managed for decades.

Standing dead (SD) trees created by tree die-off have sparked concern that fire hazard, carbon emissions, and air quality may worsen in population centers near the affected areas. Some wildland fire scientists fear that dense SD trees will lead to “mass fires” in the coming decades^10^. Mass fires are phenomena in which long-burning fuels (such as dead and downed tree trunks) burn simultaneously over a large area, creating strong fire-induced winds with the potential to damage or destroy nearby infrastructure. In light of these risks, local and state policies have favored removal of SD biomass, particularly near communities. Debate continues among scientists regarding the magnitude and nature of fire risk resulting from tree mortality, the effects of die-off and biomass energy on carbon emissions, and the impact of tree removal on the ecology of the forests^10–13^. Given these uncertainties, we do not promote a specific course of action to address SD trees but rather evaluate the feasibility of tree removal in order to inform policy objectives.

After SD trees are harvested, sustainably disposing of them is economically and logistically challenging. In California, SD tree harvests for timber are limited by scarce domestic mill infrastructure and policies restricting the export of unprocessed wood^14^. Since wood quality degrades rapidly after death, SD trees quickly lose value as timber in the initial years after death^15^. Additionally, landfills and composting are not viable options for large-scale disposal of woody biomass^14^. Though the state has worked to create low-cost applications of dead wood such as mulch and sign posts, these solutions are considered “low volume” because of their limited markets relative to the quantities of SD biomass that state agencies aim to remove^14,16^. Therefore, the primary means of removal for many SD trees of merchantable size is to burn them in open piles, which release CO_2_, particulate matter, and other criteria pollutants that adversely affect human and environmental health^17^.

Woody biomass-derived energy represents a viable option for the sustainable disposal of SD trees in California and elsewhere, in part because its emissions are cleaner than those of open pile burning^17,18^. There is a longstanding precedent for coupling biomass energy with forest management in North America^19,20^. There has even been research on electricity generation from areas that have experienced beetle-induced die-off^21^. However, woody biomass energy has declined in California in recent decades due to low electricity prices and high feedstock costs^22^. In an effort to incentivize bioenergy production, California declared a state of emergency due to tree mortality in 2015 that directed state agencies to take steps toward facilitating SD tree removal immediately^23^. One of the state’s central policy objectives is to help make woody biomass energy more economically feasible by a) funding technological development, and b) providing incentives to build new bioenergy facilities that use biomass from areas designated as tree mortality High Hazard Zones (HHZs), with particular emphasis on 10 “High Priority Counties” that contain high densities of dead trees. It is yet unknown whether widespread biomass energy using SD wood is attainable given the geographical distribution of SD biomass and its characteristics.

To evaluate the potential for biomass energy as a SD tree disposal solution, detailed information on SD biomass feedstock availability is needed^20^. SD biomass has not yet been quantified due to limitations in data availability. For instance, LiDAR has been used successfully to estimate live biomass, but studies focusing on dead biomass are rare and LiDAR is expensive to scale to the entire state^24,25^. Satellite imagery that uses pixel-scale vegetation indexes can only detect the presence of mortality but not quantities of dead trees or biomass^24^. Methodologies relying on long-term monitoring data to model normal growth and death forest cycles have limited ability to estimate feedstock potentials^26^ – even the most extensive field data campaigns measure only ~0.0017% of forested area each year^27^, which is insufficient to capture episodic events like die-off. We propose a novel method to estimate state-wide SD biomass densities and distributions based on Aerial Detection Surveys (ADS). The USFS uses ADS to monitor dead tree counts in areas experiencing tree die-off. These areas are delineated from aircraft as geospatial polygons. However, other variables critical for estimating potential feedstock quantities and costs – such as biomass densities, distances to roads, average volumes per tree, and slope of the terrain^28^ – are not included in ADS data. While previous research has used ADS data to investigate SD biomass in North America, these studies have applied simple conversion factors to estimate biomass from tree counts for all trees of a given species, ignoring the wide variation in tree sizes that occur within regions^29,30^.

The objectives of this paper are to analyze the characteristics of the biomass left over by the California die-off and to assess how much of the resulting biomass waste could be economically used as energy feedstock. We present a new methodology for estimating SD biomass using fine-scale data sources that provides precise estimates across large geographic areas. After calculating SD biomass across the state, we filter and classify that database using criteria related to harvest feasibility. We focus on SD biomass as it pertains to bioenergy as opposed to biofuel or other conversion technologies because biomass energy has been a focus of tree die-off hazard mitigation efforts in California and elsewhere^20,31,32^. Our analysis does not evaluate the economic merits nor ecological trade-offs of tree removal (e.g. wildlife habitat value of SD trees) – we instead focus on evaluating the feasibility of governmental removal goals in areas where mitigation has been deemed necessary, such as within state-designated High Hazard Zones.

Our research questions are:

1. Can a methodology be developed to rapidly and reliably assess SD biomass quantities shortly following forest die-off using existing data sources?
2. Can biomass energy feasibly be used to facilitate the removal of a majority of SD trees in state-designated high risk areas?

## Analytical Framework

The method to estimate the potential biomass feedstock from SD trees is a multistep process. The first step was to estimate dead biomass within the areas affected by tree mortality and surveyed in the ADS. Subsequent filtering of this biomass was based on criteria specific to biomass energy. We estimated SD biomass densities at fine spatial resolution by combining ADS data with the Landscape Ecology, Modeling, Mapping, and Analysis (LEMMA) Gradient Nearest Neighbor (GNN) Structure (Species-Size) Maps. We used ADS data obtained from USFS Pacific Southwest Region (USFS R5)^8^, and GNN forest structure data from the LEMMA lab, found at https://lemma.forestry.oregonstate.edu/data/structure-maps33 (See details in Supplementary Information).

The second step was to remove unrecoverable biomass from the totals using the following operational criteria: a) spatial isolation (sparse pockets of mortality can make harvesting expensive); b) wilderness designation (mechanical operations prohibited); and c) tree volume limitations (average tree volume can exceed chipping machinery capacities).

Finally, we classified the feasibly harvestable biomass into two categories: more- and less-economical. This classification was based on (a) whether average tree volume exceeds *on-site* chipping capacities, which are more restrictive than chipping capacities of machinery at biomass facilities; (b) terrain slope; and (c) distance to the nearest road. We also classified the biomass feedstock by location within HHZs Tiers 1 and 2. A more detailed breakdown of this analytical process follows below, and additional details on methodology can be found in Supplementary Information.

Note that our assumption is that most of the residues from harvesting SD trees will be chipped for bioenergy applications. In other words, the analysis is constrained to biomass-only harvests and excludes integrated operations that harvest sawlogs and biomass simultaneously. Though integrated approaches can reduce costs^20^, large-scale removal of dead biomass will most likely necessitate biomass-only harvest methods due to the large quantity of SD biomass and the timber infrastructure limitations described above.

### Calculating total SD biomass

We used GNN forest structure maps based on 2012 satellite imagery as our pre-drought baseline reference. The GNN method combines remote sensing data, Forest Inventory and Analysis (FIA) plot data, and other data sources to interpolate detailed forest structure variables across all forested land in a region at 30-m resolution^33^. Our methodology uses the Component Ratio Method^34^ (CRM) for calculating aboveground tree biomass for the feedstock estimations, which are included in the forest structure variables interpolated by GNN. We estimate uncertainty in the analysis by using an upper and lower bound depending on the assumed ADS tree size detection threshold. The results are reported in bone dry tons (BDT) instead of metric tons because it is conventional to use BDT in the woody bioenergy field. A BDT is defined as 2,000 lbs of woody material at 0% moisture in the form of fuel chips.

The methodology to merge ADS and GNN datasets is as follows:

1. For each ADS mortality polygon *p* in year *t*, we cropped and masked the GNN forest structure raster to the size and shape of the polygon. We then distributed the number of SD trees according to ADS in polygon *p*, defined as *DT*_*p,t*_, across the forested pixels in *p* based on each pixel *i*’s 2012 live tree density *TPH*_*i*,_ relative to *TPH*_*i*_ in other pixels within *p*. Discrepancies occasionally occurred in which the number of SD trees in the pixel exceeded the GNN estimate of live trees. In such cases, we limited the dead tree count *DT*_*i,t*_ to the GNN estimate of live trees, as shown in Eq. (1). Each pixel has an area of 900 m^2^, or 0.09 hectare. As a result, this conversion factor is included to convert *TPH*_*i*_ into the number of trees per pixel.
  1 $$D{T}_{i,t}=\,\min \left\{D{T}_{p,t}\frac{TP{H}_{i}}{{\sum }_{i\in p}TP{H}_{i}},TP{H}_{i}\ast 0.09\right\}$$
  This calculation assumes that all trees in a polygon *p* have an equal likelihood of mortality regardless of size, and that ADS detects all trees with equal likelihood. More details on this assumption follow below under Step 4.
2. Dead biomass per pixel per year *DBM*_*i,t*_ was estimated by multiplying *DT*_*i,t*_ by the pixel’s 2012 live tree biomass, which was calculated by dividing GNN biomass per hectare *BPH*_*i*_ by *TPH*_*i*_, as shown in Eq. (2).
  2 $$DB{M}_{i,t}=D{T}_{i,t}\frac{BP{H}_{i}}{TP{H}_{i}}$$
  Steps 1–2 were repeated for each year *t* of ADS data from 2012 to 2017 and then summed across years to calculate the cumulative dead biomass *TDBM*_*i*_ for each pixel. In some pixels, *TDBM*_*i*_ exceeded 2012 GNN live tree biomass. In such cases, *TDBM*_*i*_ was limited to 2012 GNN live biomass, which was calculated by multiplying biomass per hectare *BPH*_*i*_ by the area of the pixel in hectares (0.09 hectares per pixel) as shown in Eq. (3). This correction was required in fewer than 10% of pixels and represented a reduction of only 4% of the overall cumulative sum of feedstock.
  3 $$TDB{M}_{i}=\,\min \left\{{\sum }_{t\in T}DB{M}_{i,t},\,BP{H}_{i}\ast 0.09\right\}$$
3. The above steps rely on a threshold value for diameter at breast height (DBH) above which ADS can detect SD trees. Since trees < 20–25 cm in DBH are rarely visible from aircraft (Jeffrey Moore, personal communication, 2019), we selected DBH ≥ 25 cm as our detection size threshold. Thus, we used GNN *BPH*_*i*_ and *TPH*_*i*_, data that describe the subset of live trees with DBH ≥ 25 cm. However, in areas with low tree densities or small average tree size, ADS data may detect trees smaller than 25 cm DBH. To estimate uncertainty in ADS detection thresholds, we repeated the analysis using GNN *BPH*_*i*_ and *TPH*_*i*_ data for all trees with DBH ≥ 2.5 cm. This second analysis represents lower bound estimates because trees near 2.5 cm DBH are likely to be obstructed from view by larger trees. Moreover, one of the main drivers of the tree die-off event was bark beetles, which preferentially target larger trees^9,35^. Hence, true SD biomass densities are likely closer to our upper bound. We did not account for tree decay following mortality, which leads to reductions in wood density^36^, because research on the biomass densities of SD trees is limited^37^ and we did not expect significant biomass declines for mature trees within the 5-year window that we examined.

### Analysis of SD biomass for harvest feasibility

1. Small, scattered pockets of tree mortality are economically inefficient to harvest for biomass energy. Thus, we identified clustered pixels containing SD biomass and filtered out isolated pixels. The clustering algorithm Density Based Scan Clustering (DBSCAN)^38^ was implemented using 112 pixels as the minimum number of pixels per cluster. Given that a pixel is approximately 0.09 hectares, each cluster represent approximately 10 hectares containing SD biomass.
  The DBSCAN method requires specification of a parameter termed the ε-neighborhood, which represents the local radius for expanding clusters and serves as the upper limit to define a cluster (see details in Supplementary Information). A smaller value of ε generates more compact clusters but reduces the recoverable biomass totals. We performed a sensitivity analysis to identify an optimal ε-neighborhood value. The choice of the ε-neighborhood value was determined by considering the inflection point in the trade-off curve between biomass removed from the total and average standard distance, which is a measure of the compactness of the cluster.
2. We removed SD biomass located in land designated as wilderness or National Park since harvesting in wilderness areas is not allowed by law^39^. Though harvesting is legal within non-wilderness National Park areas, it is less common than in other land ownership designations.
3. Harvest feasibility is also affected by tree size due to equipment limitations and safety considerations, since woody biomass generally must be chipped for use in bioenergy applications. Commercial large drum whole tree chippers have a maximum in-feed size of 102–127 cm DBH^40^, which is associated with a volume per tree (VPT) of about 11.32 m^3^ (400 ft^3^) for *Pinus ponderosa* or *P. lambertiana*^41^, two of the species most affected by recent die-off^9^. Trees larger than this diameter would require pre-processing before being chipped at additional cost.
  Using data from the GNN structure maps, mean VPT per pixel VPT_i_ was estimated by dividing the GNN total volume of live trees per hectare (VPH_i_) by the number of live trees per hectare (TPH_i_) as follows in Eq. (1):
  4 $$VP{T}_{i}=\frac{VP{H}_{i}}{TP{H}_{i}}$$
  Pixels with VPT_i_ ≥ 11.32 m^3^ were removed.
4. Dead tree density also affects harvest feasibility. Areas with very low tree densities can be costly to harvest, and if dead tree density is low but live tree density is high, selective removal of dead trees could be difficult due to the need to navigate around live trees. We excluded areas with very low dead tree density (<2.5 dead trees per ha).

### Classifying SD biomass by factors related to expected harvest cost

After filtering for harvest feasibility, we classified the remaining pixels in the database based on factors that are likely to influence harvest costs, including whether mean VPT exceeds on-site chipping capacities (as opposed to chipping capacities of biomass facilities) and whether slope exceeds tractor thinning capacities. This process differs from the filtering described above in that it differentiates between places where biomass harvest is more or less likely, rather than eliminating areas where it is nearly impossible. For example, harvesting in protected wilderness areas violates existing regulations, whereas harvesting on steep slopes is expensive but possible. The classification process used here does not include all factors that may contribute to harvest feasibility (e.g. road curve radii). Therefore, the results represent an upper estimate of feasibly harvestable biomass for bioenergy.

1. We classified feasibly harvestable pixels into two mean VPT classes, ≥2.26 m^3^ (∼80 ft^3^) and ≤2.26 m^3^. We based this cutoff value on the maximum volume that can be processed on-site with commercial mechanical equipment^19,42^. Trees with volumes ≥2.26 m^3^ must be cut into logs and chipped at sawmills or chipping facilities, which may increase costs. Note that earlier we excluded pixels where VPT ≥ 11.32 m^3^ – the maximum volume that sawmills and chipping facilities can process.
2. Each pixel was then classified based on local terrain slope. Terrain slope strongly affects the cost of harvest, primarily because it determines whether ground-based, cable-yarding, or helicopter-yarding harvest systems may be used^42^. Ground-based systems are used where conditions allow because they are typically less expensive and cause less incidental damage than cable- or helicopter-yarding^43^. For safety and environmental reasons, however, ground-based systems are generally used only when the slope is ≤40%^44^. Trees located in areas with slopes in the 30–40% range can employ either ground-based systems or cable-yarding, depending on other factors such as tree size^42^. We calculated slope values using the National Elevation Dataset (NED) as described in Supplementary Information.
3. We classified pixels based on linear distance to the nearest road. We combined road data from OpenStreetMap (https://www.openstreetmap.org) and the US Forest Service (https://data.fs.usda.gov/geodata/edw/datasets.php), and generated 2,000 m buffers around each road (described in Supplementary Information). We overlaid the resulting buffers with the pixels to classify the pixels by proximity to a road. We selected the distance cut-off based on upper estimates of maximum yarding distances for cable harvesting^45^.
4. Finally, we classified each feasibly harvestable SD biomass pixel based on whether it falls within HHZ Tier 1 or 2. State agencies delineate these priority areas for tree removal and provide certain economic incentives for biomass energy production. For instance, the Bioenergy Market Adjusting Tariff (BioMAT) feed-in tariff applies only to biomass removed from High Hazard Zones^46^.

### Calculating energy conversions

To calculate energy conversions, we made the following assumptions: bone-dry biomass has a calorific value between 18 and 22 GJ/1000 kg; chips have a 30% relative moisture content; and commercial boiler technology used for electricity generation operates at 120 °C and a thermal efficiency of 90%^47^. Based on these assumptions, we calculated a conversion rate of approximately one bone-dry ton (BDT) biomass per MWh (1 BDT/MWh), which is consistent with industry values^48,49^.

In addition to conventional boiler technology, the state has favored small and medium scale biomass gasification because it enables facility siting closer to forest biomass supplies^50,51^. For small-scale gasification technology, conversion efficiencies from woody biomass to electricity are lower than for boilers and are highly variable, ranging between 12% and 40%^52^. In this study, we assume a gasifier- electricity generator system of 150 kW with a conversion factor of approximately 4.7 BDT/MWh^53^.

### Validation

In order to validate our methodology of SD biomass estimation, we compared our estimates of SD tree biomass to a separate data set generated using field and remote sensing data sources rather than ADS. This data set, called LT-GNN, combines Landtrendr with gradient nearest neighbor interpolation of raw FIA data across all forested pixels in California^54^. LT-GNN was applied to California forests annually through 2016. By subtracting the 2016 LT-GNN biomass raster from that of 2012, we generated alternative estimates of biomass loss against which to compare our ADS-based methodology. We constrained our biomass loss estimates to 2012–2016 for the comparison rather than 2012–2017 because LT-GNN results are not available for 2017. We also constrained the comparison to areas where ADS detected mortality.

For each ADS polygon, we summed 2012–2016 biomass loss according to the LT-GNN results, excluding pixels where LT-GNN predicted net biomass gain (from growth) rather than loss, and compared this sum to our 2012–2016 cumulative SD biomass estimate. We compared polygons that met minimum harvest feasibility requirements according to this study, namely mean VPT < 11.3 m^3^, dead tree density >2.5 trees per ha, and not spatially isolated according to our DBSCAN clustering method. Finally, we excluded polygons smaller than 2700 m^3^, or three LT-GNN pixels, because of inaccuracies in aligning polygons with raster pixels at such small polygon sizes.

We calculated root mean square error (RMSE) between the two methodologies, including both upper bound and lower bound estimates generated in this study. Because of high heteroscedasticity of the results, with higher variation at higher values, we applied the RMSE calculation to log-transformed biomass estimates.

## Results

### Statewide SD tree biomass resulting from forest die-off

**Figure 1:**
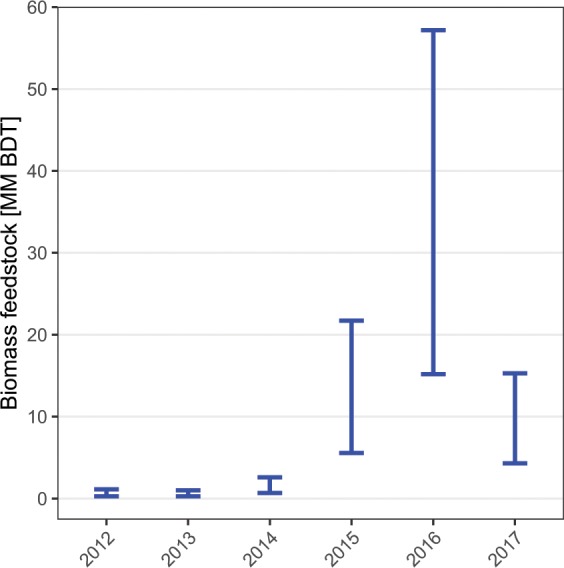
Dead biomass in California by year of detection. The upper bound estimates, represented by the upper bars, were calculated using the assumption that ADS detects all dead trees ≥25 cm DBH with equal likelihood. The lower bound estimates, represented by the lower bars, were calculated using the assumption that ADS detect all trees ≥2.5 cm DBH with equal likelihood. Biomass is reported in million (MM) BDT. Yearly biomass estimates displayed have not been corrected in instances where 2012–2017 dead biomass estimates exceed 2012 GNN live biomass because it is impossible to parse that correction across individual years.

Between 2012 and 2017, approximately 95.1 million BDT of SD biomass resulted from tree mortality, with a lower bound of 26.2 million BDT. The most significant increase in SD biomass occurred in 2016 (Fig. 1). We found that 1.3–4.8% of the aboveground tree biomass across all forested land in California that was alive in 2012 died by 2017.

**Figure 2:**
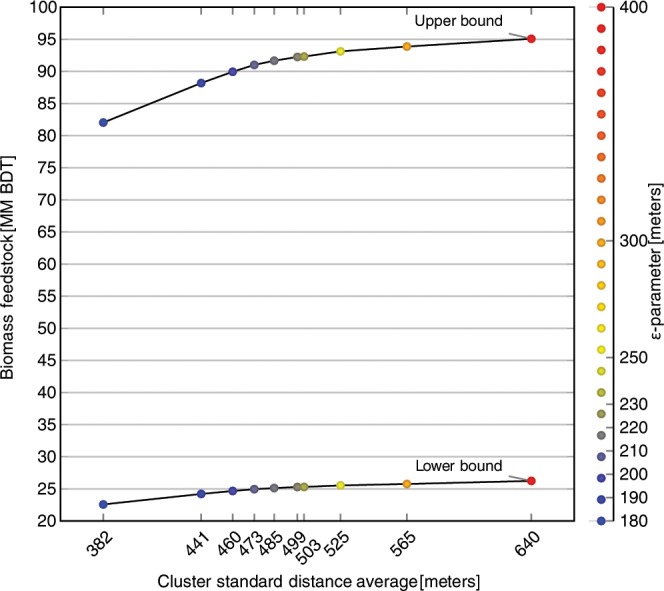
The trade-off curve between reduction in total SD biomass and cluster compactness measured by average standard distance after performing DBSCAN clustering using a range of ε-values and eliminating isolated pixels. The upper curve represents biomass estimates using the assumption that ADS detects all dead trees ≥25 cm DBH with equal likelihood, while the lower curve represent the lower bound estimate made using the assumption that ADS detects all trees ≥2.5 cm DBH with equal likelihood. The final ε-value used in DBSCAN clustering was chosen by locating inflection points along these curves, with a result of ε = 220. The DBSCAN algorithm does not generate clusters using ε-values < 180 because the minimum pixel count of 112 cannot be reached within a radius < 180 m. On the other hand, DBSCAN clustering using ε-values > 400 does not achieve the goal of filtering out scattered SD biomass that would be inefficient to harvest. Biomass is reported in million (MM) BDT.

The sensitivity analysis used to determine the most effective filtering of sparse pockets of biomass found an inflection point in the trade-off curve between filtered biomass and cluster compactness at ε = 220 (Fig. 2). Filtering out spatially isolated pixels using an ε-neighborhood parameter of 220 reduced the statewide SD biomass totals by 1.0–2.7 million BDT, to a range of 25.3–92.3 million BDT (Table 1).

**Table 1:** Biomass reduction from sequential filtering.

Filter	Lower bound biomass reduction (million BDT)	New lower bound total (million BDT)	Upper bound biomass reduction (million BDT)	New upper bound total (million BDT)
Spatial isolation	1.0	25.3	2.7	92.3
Wilderness/National Parks	5.8	19.5	19.9	72.4
VPT_i_ ≥ 11.32 m^3^ or <2.5 dead tree per ha	1.1	**18.4**	3.4	**68.9**

Bold values show the final “feasible biomass” quantities used for subsequent analyses.

**Figure 3:**
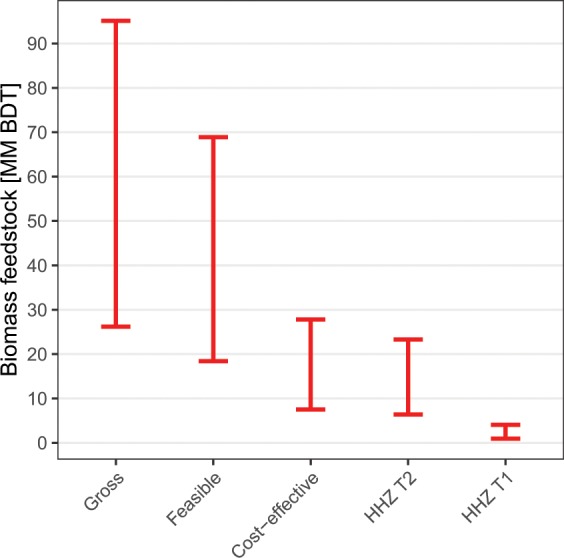
Total SD biomass that has resulted from 2012–2017 tree mortality in California. “Gross” values show total SD biomass. “Feasible” bars show the subset of SD biomass lacking characteristics likely to impede harvest feasibility, including geographic isolation, wilderness or National Park designation, and tree volumes exceeding standard chipping capacities for either on-site or facility-based chipping. “Cost-effective” bars show the subset of “Feasible” biomass that is in areas with slopes < 40% and mean tree volumes < 2.26 m^3^, where chipping can occur on-site. “HHZ T2” and “HHZ T1” columns show the subsets of cost-effective biomass located within Tier 2 and Tier 1 High Hazard Zones, respectively. The upper bars represent biomass estimates using the assumption that ADS detects all dead trees ≥25 cm DBH with equal likelihood, while the lower bars represent estimates made using the assumption that ADS detects all trees ≥2.5 cm DBH with equal likelihood. Filtering for harvest feasibility (upper bound) resulted in a 30% reduction in the available feedstock and filtering for cost-effectiveness resulted in a 70% reduction from Gross. Biomass is reported in million (MM) BDT.

Filtering the remaining areas by wilderness/National Park status, tree sizes, and low dead tree densities (Table 1) caused a further reduction of the total feasible biomass to a range of 18.4–68.9 million BDT (Fig. 3).

**Figure 4:**
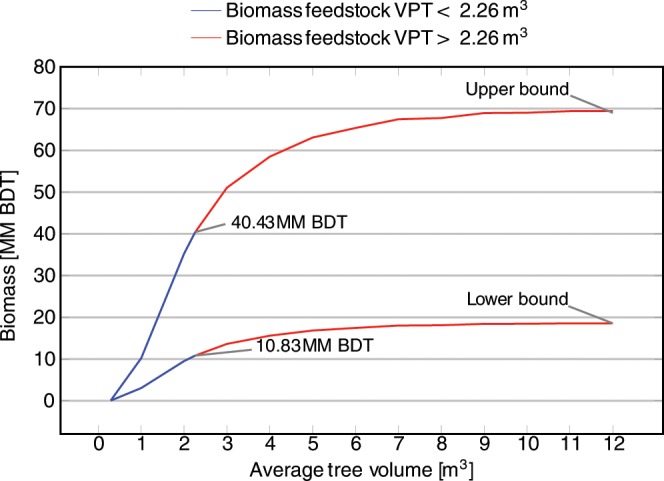
Running cumulative sums of statewide SD biomass with respect to mean VPT per pixel. The upper curve represents biomass estimated using the assumption that ADS detects all dead trees ≥25 cm DBH with equal likelihood, while the lower curve represent the lower bound estimate made using the assumption that ADS detects all trees ≥2.5 cm DBH with equal likelihood. Below 2.26 m^3^ VPT, biomass can be chipped on-site (blue), whereas above 2.26 m^3^ trees must be chipped at facilities before further processing (red). There are a total of 40.4 million BDT SD biomass, with a lower bound of 10.8 million BDT, in areas where the average VPT is <2.26 m^3^. This represents less than 60% of the total feedstock available. Biomass is reported in million (MM) BDT.

**Figure 5:**
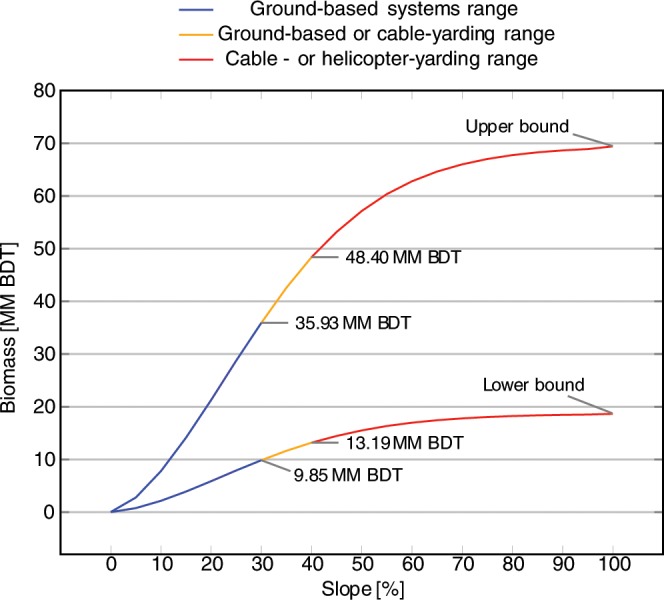
Running cumulative sums of statewide SD biomass with respect to slope. The upper curve represents biomass estimates using the assumption that ADS detects all dead trees ≥25 cm DBH with equal likelihood (Upper bound), while the lower curve represent the lower bound estimate made using the assumption that ADS detects all trees ≥2.5 cm DBH with equal likelihood (Lower Bound). Below 30% slope, ground-based harvesting can be used (blue), whereas in terrain with slopes 30%-40%, either ground-based or cable-yarding systems may be used depending on other factors (yellow). Above 40% slope, cable- or helicopter-yarding systems must be used (red).

The results of classification by cost-effectiveness criteria are summarized in Table 2. Over 60% of the SD tree biomass can be chipped on site (Fig. 4). Areas with slopes <30% contain 9.9–35.9 million BDT biomass, while areas with 30–40% slopes contained 3.3–12.5 million BDT (Fig. 5). We defined a subset of feasibly harvestable biomass as “cost effective” based on the following criteria: tree volumes small enough to chip onsite, SD trees located in areas with slope < 40%, and SD trees ≤2,000 m from roads.

**Table 2:** Classification of feasible biomass based on each cost-effectiveness criterion.

Factor	Lower bound not cost-effective (million BDT)	Lower bound cost-effective (million BDT)	Upper bound not cost-effective (million BDT)	Upper bound cost-effective (million BDT)
On-site chipping (VPT ≤ 2.26 m^3^)	7.8	10.8	29.0	40.4
Ground-based harvesting (slope < 40%)	5.8	12.6	21.6	47.3
Accessibility (<2000 m from road)	0.4	18.0	1.4	67.5

**Figure 6:**
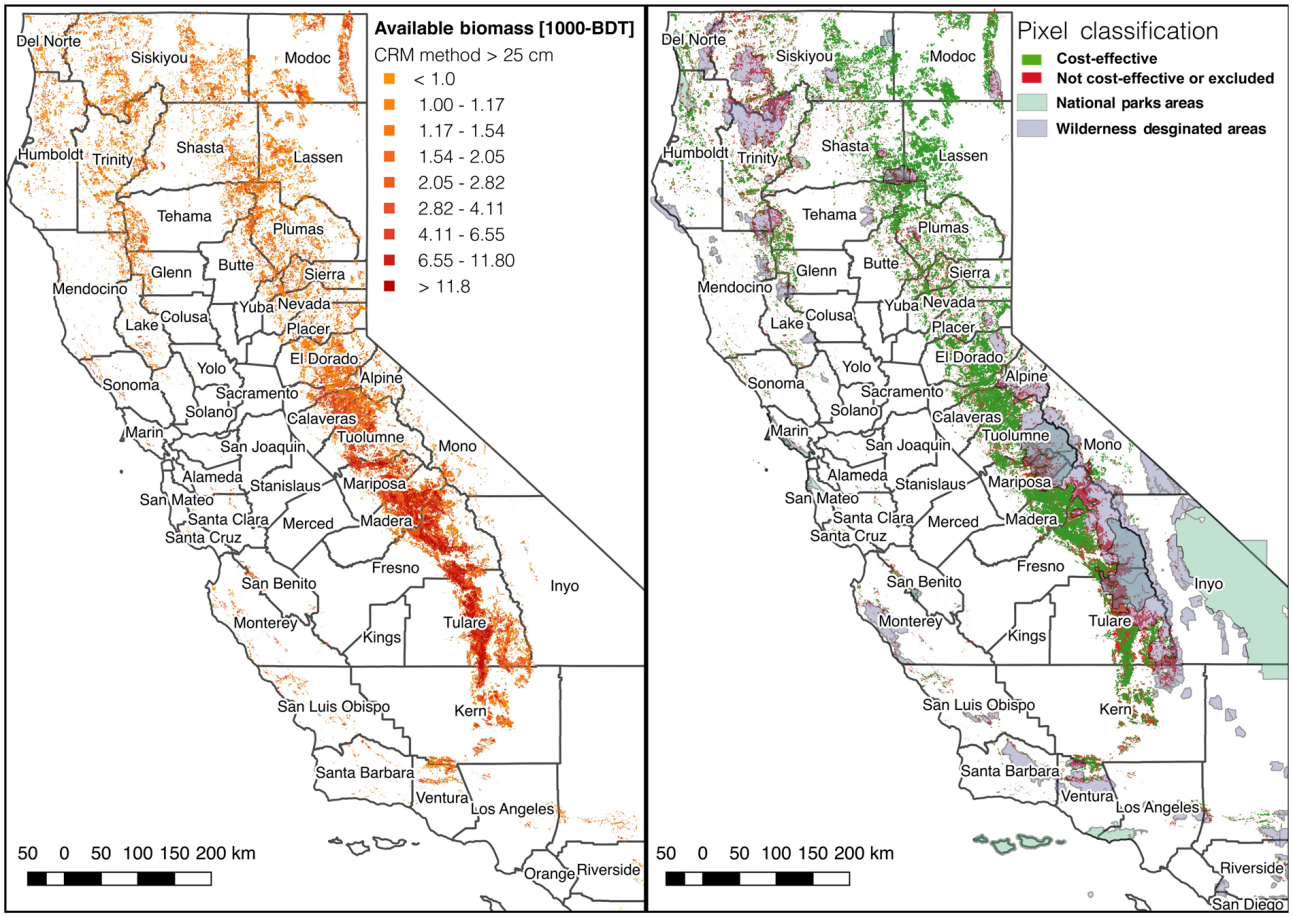
Dead aboveground tree biomass resulting from tree die-off in 2012–2017. (**a**) Dead aboveground tree biomass (upper bound estimates assuming ADS detection of trees >25 cm DBH) resulting from tree die-off in 2012–2017 in 1000s of bone-dry tons (BDTs). (**b**) Comparison of cost-effective areas of SD biomass and economically infeasible areas. Black lines represent county boundaries. Map was produced from results generated in this study using QGIS 3.8.3 (https://qgis.org/en/site/forusers/download.html). Supporting data are described in Supplementary Information.

The resulting subset amounts to 7.5–27.8 million BDT. Given that recent policies specifically target SD tree removal within HHZs, we also classified both feasible and cost-effective biomass by HHZ (Table 3, Fig. 3). The HHZ Tier 2 areas encompass most of the feasible and cost-effective biomass. Dead tree biomass is concentrated in only 12 of California’s 58 counties, most of which are in the southern Sierra Nevada (Fig. 6 and Table 4). County-level estimates of SD biomass by HHZ tier are listed in Table 5 for the counties with highest SD biomass and in Table S2 for all counties.

**Table 3:** Classification of feasible and cost-effective SD biomass by High Hazard Zone (HHZ).

HHZ Tier	Lower bound feasible (million BDT)	Upper bound feasible (million BDT)	Lower bound cost-effective (million BDT)	Upper bound cost-effective (million BDT)
Tier 1	2.0	8.3	1.0	4.1
Tier 2	15.4	57.2	6.4	23.3
Total in HHZ	15.6	58.0	6.5	23.8
No HHZ	2.8	10.9	1.1	4.0

Note that there is overlap between tier designations of HHZ such that Tier 1 and Tier 2 biomass quantities sum to more than the total across both tiers.

**Table 4:** SD biomass in the counties most affected by 2012–2017 tree die-off*.

County	Gross [1000 BDT]		Feasible [1000 BDT]		Cost-effective [1000 BDT]		Cost-effective proportion of Gross [%]	
	DBH > 25 cm (UB)	DBH > 2.5 cm (LB)	DBH > 25 cm (UB)	DBH > 2.5 cm (LB)	DBH > 25 cm (UB)	DBH > 2.5 cm (LB)	DBH > 25 cm (UB)	DBH > 2.5 cm (LB)
Tulare^†^	22,866	6,739	12,619	3,634	3,379	1,004	14.78	14.89
Fresno^†^	15,137	4,701	11,410	557.8	3,924	1,287	25.92	27.37
Madera^†^	10,134	3,192	8,595	2,714	3,452	1,079	34.06	33.79
Tuolumne^†^	7,734	1,879	6,339	1,492	2,581	635	33.37	33.80
Mariposa^†^	7,401	1,883	4,480	1,010	1,847	422	24.95	22.43
Kern^†^	3,673	1,072	3,220	912	1,189	345	32.38	32.15
Siskiyou	3,483	895	2,579	639	1,215	312	34.87	34.89
Calaveras^†^	2,468	535	2,365	509	1,027	231	41.60	43.27
El Dorado^†^	2,392	601	2,230	552	1,066	273	44.58	45.40
Modoc	2,355	706	2,191	641	1,744	502	74.05	71.12
Plumas	2,089	485	1,809	409	880	209	42.11	43.17
Lassen	1,414	341	1,258	293	1,093	255	77.30	74.88
Total	81,146	23,028	59,094	16,362	23,396	6,555		

*See Supplementary Tables S2 and S3 for SD biomass results for all counties.

^†^These counties have been identified as High Priority Counties by the state of California.

The column “Gross Total” lists SD biomass before filtering. The column “Feasible” lists SD biomass after filtering for scattered pixels, wilderness/National Parks, mean VPT > 11.32 m3, and dead trees per ha < 2.5. The column “Cost Effective Total” lists feasibly harvestable SD biomass available at slopes < 40%, average tree volumes < 2.26 m^3^ and within 2000 meters from the roads.

**Table 5:** SD biomass in the counties most affected by 2012–2017 tree die-off, by HHZ tier, after filtering for harvest feasibility and cost-effectiveness.

County	Feasible [1000 BDT]		Cost-effective [1000 BDT]	
	HHZ Tier 1	HHZ Tier 2	HHZ Tier 1	HHZ Tier 2
Tulare	1,072.4	9,640.2	459.2	2,449.2
Fresno	496.8	9,777.1	200.5	3,519.6
Madera	683.1	8,121.0	385.7	3,255.5
Tuolumne	1,219.7	6,012.3	563.1	2,451.9
Mariposa	842.5	3,706.7	432.1	1,589.4
Kern	327.1	2,013.3	144.9	701.7
Siskiyou	154.8	1,988.6	92.8	1,000.7
Calaveras	1,017.1	2,308.1	482.6	1,002.4
El Dorado	318.8	1,853.7	158.6	898.1
Modoc	175.9	1,882.9	152.4	1,487.9
Plumas	291.5	1,568.8	164.3	756.6
Lassen	86.1	1,136.0	72.9	983.7

The column “Feasible” lists SD biomass after filtering for scattered pixels, wilderness/National Parks, VPT > 11.32 m^3^, and dead trees per ha < 2.5. The column “Cost Effective Total” lists feasibly harvestable SD biomass available at slopes < 40%, average tree volumes < 2.26 m^3^ and within 2,000 meters of roads.

### Validation

**Figure 7:**
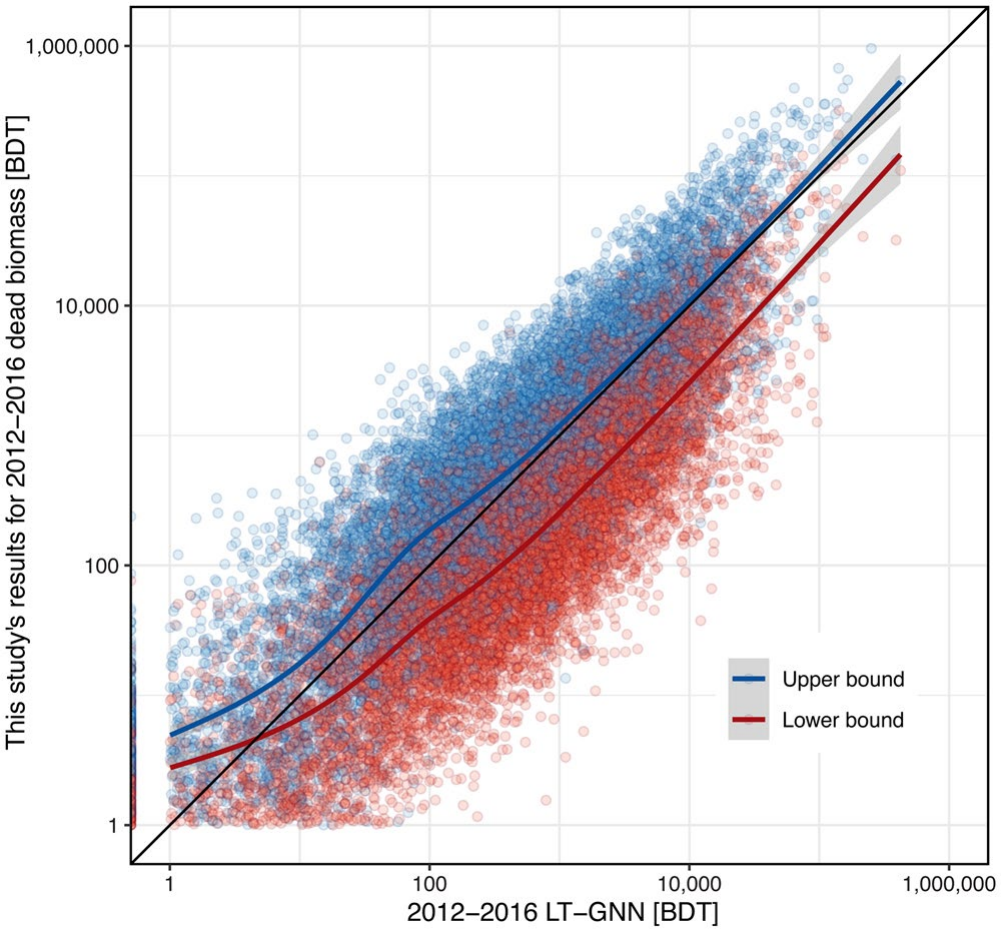
Comparison of this study’s results with biomass loss estimates generated from 2012–2016 LT-GNN data. Each point represents one ADS polygon. The black line represents a 1:1 fit, while the blue and red lines represent generalized additive model fits for the upper and lower bounds, respectively, which were modeled using cubic splines according to the function “geom_smooth” in the R package “ggplot2.” Gray shading represents 95% confidence intervals.

On average, biomass loss estimated by the LT-GNN method fell between our upper and lower bound biomass estimates (Fig. 7). As expected, our upper bound estimates performed better on average than our lower bound estimates. For the upper bound estimates, RMSE on a log scale was 0.53, while RMSE for the lower bound was 0.74. Our method predicts higher biomass loss than LT-GNN in low-mortality areas, likely because we limited the comparison to locations with positive mortality according to ADS.

### Energy potential and example use cases

Assuming conventional boiler technology, feasibly harvestable biomass has the potential to produce 18.4–68.9 TWh of electricity. On the other hand, using small-scale gasification technology, the potential electricity production would be 4.3–13.8 TWh. If electricity production is assessed only considering cost-effective SD biomass, the energy potential is 7.5–27.8 TWh with conventional boilers and 1.7–6.4 TWh using small scale gasification. For context, California generated a total of 198 TWh in-state energy of all types in 2016 (Supplementary Table S7).

California’s BioMAT program incentivizes energy using byproducts of sustainable forest management and requires that most of the feedstock be sourced from HHZs. The program has a deployment target of 50 MW (http://www.cpuc.ca.gov/sb_1122/). If the state meets that target, there is enough feasible biomass to supply conventional power plants in HHZ for 39–145 years at an 80% capacity factor (0.4 TWh/year), and enough cost effective biomass to supply plants for 16–60 years.

These results can be applied to facility siting as well as feedstock analysis for existing biomass energy plants. For example, the Pacific Ultrapower Chinese Station (PUCS) is a biomass power plant located in Central California. The facility, which has a 25 MW production capacity, was built to utilize woody biomass from forest management operations and could potentially be used to process SD trees.

**Figure 8:**
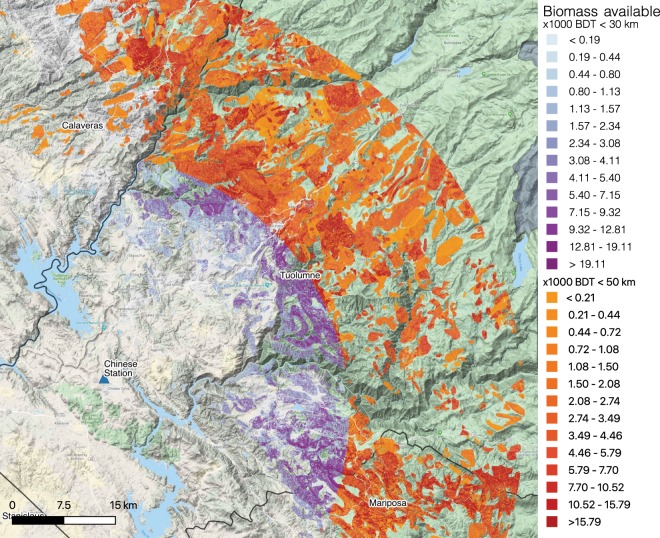
SD biomass within 30 km (purple) and 50 km (red) of Pacific Ultrapower Chinese Station. Map was produced from results generated in this study using QGIS 3.8.3 (https://qgis.org/en/site/forusers/download.html). Supporting data are described in Supplementary Information.

Using the database and analytical framework created from this study, we estimated that the feasibly harvestable biomass feedstock available to PUCS is 0.4–1.6 million BDT within a 30 km radius and 1.3–6.1 million BDT within a 50 km radius (Fig. 8). Approximately 1.1 million BDT within 30 km (lower bound 0.3 million BDT) and 4.3 million BDT within 50 km (lower bound 1.0 million BDT) of potential feedstock near PUCS are located in terrain with slope <40%, enabling the likely use of ground-based harvesting systems for tree removal. In terms of cost-effective supply, there are 0.2–0.7 million BDT and 0.6–2.5 million BDT of feedstock within 30 and 50 km, respectively, of PUCS.

PUCS can produce approximately 0.2 TWh of energy per year, assuming an 80% capacity factor with a conventional boiler. Therefore, there is sufficient cost-effective feedstock for electricity generation potential of 200–700 GWh within a 30 km radius of the facility, equivalent to 1–4 years of operation if the plant relied only on SD biomass. Within the 50 km radius, the potential increases to 600–2500 GWh, which translates to 3–14 years of operation.

## Discussion

### Evaluation of analytical framework

This study’s geographically explicit assessment of SD biomass enables detailed estimations of woody feedstock availability resulting from tree die-off. While the results are specific to California, the analytical framework provides a general approach applicable to forests worldwide that have experienced tree die-off. By combining aerial detection surveys with forest structure maps, we found that 18.4–68.9 million BDT of SD tree biomass could feasibly be used as energy feedstock in California, with the true value likely closer to the upper bound of that range.

Our validation procedure demonstrated that an independent, more time-intensive methodology for estimating the biomass of recent tree mortality within ADS survey areas found similar magnitudes of mortality to our upper bound estimates. Because of the better alignment of upper-bound results with LT-GNN than lower-bound results, we refer only to upper bound results in the remainder of the discussion. Although our comparison with LT-GNN allows us to validate the methodology used in this paper and highlight the value of ADS data, the LT-GNN data do not represent metrics identical to those produced in our study: they represent forest biomass stocks (incorporating both growth and mortality) whereas our method estimates the flux in standing dead biomass (representing only mortality); they do not include 2017 mortality; and they capture both background mortality and insect- and drought-mediated die-off, whereas our method, through its use of ADS data, captures the effect of die-off in particular. Nevertheless, validation of our results against LT-GNN helped to show that the methodology introduced in this paper provides a reliable means of rapidly assessing SD biomass densities after die-off. We used readily available data sources that were either available prior to die-off (GNN maps) or gathered yearly and made publicly available only months after data collection (ADS). Going forward, real time monitoring of tree die-off can improve by combining insights from aircraft surveys (i.e. ADS) with more timely information on forest structure obtained from satellite sensors (e.g., GNN).

### Feasibility of widespread biomass energy using SD trees

Based on these findings, harvesting SD trees for energy feedstock is not a comprehensive solution for disposing of a majority of recently dead trees in California deemed for removal, nor for disposing of a majority of SD trees in areas of highest priority to the state of California. Of the 95.1 million BDT of SD biomass evaluated, only 29% may be cost-effective to recover. In HHZ Tiers 1 and 2, ~42% and ~30% of SD biomass, respectively, are cost-effective.

The major factors contributing to high costs of SD tree removal were tree volume and slope. Filtering for feasibility did not strongly affect SD biomass quantities where mitigation is targeted. Although filtering out wilderness and National Park areas reduced feasible biomass by over 25%, these areas do not contain at-risk communities nor infrastructure, making them low priorities for mitigation. Filtering for spatial isolation and volumes larger than off-site chipping capacities reduced feasible biomass by only 2.8% and 4.7%, respectively. Slope and tree volume played the largest roles in classification by cost-effectiveness. Only 58% of feasibly harvestable biomass met VPT criteria for on-site chipping and only 69% of feasibly harvestable biomass met slope criteria for lower-cost ground-based harvesting systems. Accessibility had relatively little impact on cost-effective biomass – 98% of feasibly harvestable biomass was located within 2000 m of a road.

The accessibility analysis performed here does not include road distances to existing biomass facilities because this study is designed to inform, in part, the construction of new facilities. An analysis of hauling distances and transportation costs would further reduce the quantity of economically viability biomass in California, as transportation is a major contributor to woody biomass feedstock costs^55^. Future research in this area, perhaps applying a techno-economic analysis that incorporates our database, would provide useful information on the cost-effectiveness of biomass energy relying on existing facilities.

Dead tree biomass is concentrated in only 12 of California’s 58 counties, most of which are in the southern Sierra Nevada (Fig. 8 and Table 4). There is incomplete overlap between these 12 counties and the ten counties designated as High Priority according to the California state government (Table 4), likely because the state considered dead tree density, rather than dead tree biomass, as the criterion for “severe mortality” (see https://fmtf.fire.ca.gov/working-groups/tree-mortality). The ten High Priority counties receive greater state resources for tree mortality response and mitigation than other counties. In some counties with large amounts of SD biomass, the proportion of SD biomass that may be cost-effective for biomass energy is much lower than the statewide average. This finding highlights the financial challenges of using energy conversion to handle residues from tree removal. For example, in Tulare County, which has the highest gross SD biomass and is a High Priority County, cost-effective SD biomass accounts for only ~14.8% of the total (Table 4).

Nevertheless, harvestable biomass could produce a substantial quantity of electricity. Our estimate of state-wide “cost-effective” SD biomass is equivalent to 4–14% of California’s annual in-state electricity generation (see Supplementary Table S7). In our example case study, the PUCS power plant could run for several years if it used only biomass from cost-effectively harvestable SD trees.

### Implications for carbon accounting

Our results will also aid in the statewide carbon accounting needed for California to reach its climate change mitigation goals. Specifically, this study provides the first estimate of live tree carbon loss resulting from the 2012–2015 California drought. Our estimates of total SD biomass are equivalent to 11.2–40.6 Tg C across all areas surveyed by ADS, or roughly 1.1–3.8% of total aboveground forest carbon in California^56^. As SD trees decay or burn over time, their carbon will transition to the atmospheric pool. Though carbon release via decay or wildfire may take decades, carbon release from trees that are removed for safety concerns and pile burned is more likely to occur in the short term. The SD trees most prioritized for removal, namely those in Tier 1 HHZs that meet feasibility constraints, contain 0.9–3.5 Tg C, while feasibly harvestable SD trees in Tier 2 HHZs contain 6.6–24.4 Tg C. In order to understand how tree mortality may affect climate change, more research is needed on how SD tree carbon is released over time as greenhouse gases with and without tree removal.

### Conclusion

This study’s findings highlight the potential for rapid, reliable biomass assessments following forest die-off. While ADS data are often made available within months after mortality, more computationally intensive assessments such as LT-GNN can take years to process and can be difficult to acquire in detail due to confidentiality limitations on the raw data they use (namely, the Forest Inventory and Analysis Phase 2 plot inventories). The methodology outlined here could help inform mortality mitigation strategies within a time frame that allows for tree removal before treefall and decay make mitigation more difficult.

Our findings highlight the challenges in managing and responding to large-scale tree die-off and the complexities of disposing of biomass residues through energy conversion. Even under the most optimistic assumptions, woody biomass energy is unlikely to provide a widespread means of SD disposal in California. State policies to address risk in high-mortality counties may need a more comprehensive approach that includes other SD disposal methods or direct feedstock subsidies in addition to electricity generation.

## Supplementary information

Supplementary Information.

## Data Availability

The data that support the findings of this study and the resulting SQL file to restore the database are available in *figshare* at 10.6084/m9.figshare.c.4117328.v2. Statewide SD biomass results can also be downloaded from an interactive web map at http://geodata.ucanr.edu/biomass/.
