# Supplementary material for: Characterization of the woody biomass feedstock potential resulting from California’s drought
Source: Sci Rep. 2020 Jan 23;10:1096. doi: 10.1038/s41598-020-57904-z (PMC6978512; doi:10.1038/s41598-020-57904-z)
Supplement: Supplementary file 1 — Supplementary Information. [file 41598_2020_57904_MOESM1_ESM.pdf]

Supplementary Information

Characterization of the woody biomass feedstock potential resulting from California's drought

Carmen L. Tubbesing*[1], Jose Daniel Lara[2,3], John J. Battles[1], Peter W. Tittmman[4], Daniel M. Kammen[2,3,5]

[1]Department of Environmental Science, Policy and Management, UC Berkeley

[2]Energy and Resources Group, UC Berkeley

[3]Renewable and Appropriate Energy Laboratory

[4]Forest Products Laboratory, UC Berkeley

[5]Goldman School of Public Policy, UC Berkeley

*Correspondence and requests for materials should be addressed to C.L.T. (email: ctubbesing@berkeley.edu)

Data Sources

Aerial Detection Surveys

Aerial Detection Survey (ADS) data was obtained from the US Forest Service (USFS) Pacific Southwest Region[1]. ADS data is gathered annually to monitor tree mortality and damage across the state. Surveys are conducted from small aircraft on a 4-mile grid across the majority of forested land in California, including all National Forests, National and State Parks, and most forested private land[2]. However, exact coverage varies slightly from year to year. From small aircraft, highly trained observers manually record mortality that occurred in the previous year as polygons onto digital aerial imagery in computer touch tablets. Mortality is defined as standing dead trees that have died since the last survey[3]. For relatively small pockets of mortality, the concentration of mortality in each ADS polygon is expressed as the total number of dead trees. For larger polygons, mortality is recorded as trees per acre, which is then scaled up to total number of dead trees using the size of the polygon[4]. Generally, areas with < 1 dead tree per acre are considered to have "background" or "normal" levels of mortality and are not usually mapped during the flight, unless low levels of mortality are indicative of a localized pest-related event. Areas with mortality in excess of background levels are mapped to the finest resolution practicable. We used

ADS mortality data, ignoring data on non-mortality damage. In 2017, the ADS methodology changed, such that ADS observers now classify mortality density into 5 categories based on percent mortality (1-3, 3-10, 10-30, 30-50, 50+). To match the format of previous years' data, these categories are converted to TPA and number of trees based on expert judgement and the range of TPA mortality values from previous years. Thus, ADS mortality estimates from 2017 are less precise than those of 2012-2016.

Forest GNN Structure (Species-Size) Maps

The Gradient Nearest Neighbor (GNN) Structure (Species-Size) Map was obtained from the Oregon State University Landscape Ecology, Modeling, Mapping, and Analysis (LEMMA) research group website[5]. This map was developed using the gradient nearest neighbor (GNN) interpolation method to assign forest structure and species attributes from Forest Inventory and Analysis (FIA) data to each pixel at 30m x 30m resolution. The FIA program is a USFS monitoring effort that measures field plots on a 6,000 acre (2,428 hectare) grid across all forested land in California every 5-10 years [6]. GNN relies on the statistical relationship between FIA field measurements and Landsat-derived inputs as well as other predictor variables (i.e., climate, topography, parent material and location)[7,8]. The GNN forest structure maps provide critical data for energy feedstock calculations including tree density (TPH) and aboveground live biomass per hectare (BPH) calculated using two different methods: the component ratio method (CRM)[9] and a more simplified method, referred to as the Jenkins method. We used CRM biomass density estimates in our analyses. Digital GNN maps are provided as 30-m-resolution rasters in which each grid value is a unique plot number that links to the plot database containing detailed vegetation data. The present study uses GNN maps developed in 2014 using 2012 satellite imagery. The GNN models apply only to forest land, defined as areas currently supporting or with the potential to support at least 10% tree cover.

Elevation data and slope estimation

Elevation data was obtained from the US Geological Survey National Elevation Data (NED), which is derived from diverse source data that are processed to a common coordinate system and unit of vertical measure. The data are available at: https://lta.cr.usgs.gov/NED.

GDAL was used to merge all of the National Elevation Data (NED) tiles for the state of California and calculate slopes. The most relevant slope for harvest operations is the slope over the distance logs need to be yarded. In order to estimate this, the slope map generated from NED tiles was resampled with the

command `gdal_translate`, setting the outsize parameter at 5%. This calculated the average slope over areas 3.4 hectares in size (207x162 meters).

Sensitivity analysis to filter scattered biomass

We performed a sensitivity analysis to identify the optimal ε-neighborhood value. For each possible value of ε, the amount of scattered biomass that would be removed from the statewide total was calculated and compared to the average standard distance. Standard distance is a measure of the compactness of a cluster, and thus assesses its quality by determining the dispersion among members of a cluster[10]. In other words, to belong to a cluster, a pixel must have non-zero biomass density and be located close to many other pixels.

Filtering by distance to road

The road data was obtained from OpenStreetMaps following the procedure described in (https://www.qgistutorials.com/en/docs/downloading_osm_data.html). The resulting vector data set was buffered for 400 and 2000 meters using the `ST_Buffer` command in PostGIS. The command `ST_CoveredBy` was used to determine if the pixel was within the buffered area.

Software

PostGIS

The database created in this study was built and developed using the object-relational system PostreSQL and the geospatial analytics extension PostGIS, for which the documentation is available at https://postgis.net.The clustering command used to develop the database is `ST_ClusterDBSCAN`.

GDAL

The manipulation of raster data was done using the Geospatial Data Abstraction Library (GDAL). GDAL is a computer software library for reading and writing raster and vector geospatial data formats. Two main commands were used: 1) `gdaldem`, used to calculate the slope values from elevation data, for which the documentation is available at: http://www.gdal.org/gdaldem.html, and 2) `gdal_translate`, used to average the slope values, for which the documentation is available at: http://www.gdal.org/gdal_translate.html.

DBSCAN

The Density Based Scan (DBSCAN) clustering method is a clustering algorithm based on the notion of density, where clusters are defined as sets of points that lie inside or on the border of high-density regions in spatial databases. Given a set of points in space, DBSCAN clusters points together that have many nearby neighbors, marking as outliers points in low-density regions. The algorithm shows good performance on large spatial databases with clusters of arbitrary shapes. The principle behind the method is that a point is density-reachable from another point if it is within ε distance from a cluster of points larger than the minimum number of elements that constitute a cluster. Once a point is considered density-reachable, it is added to the closest cluster[10].

R

Calculations of SD biomass densities combining GNN forest structure maps and ADS data were performed in R 3.4.3. Comparisons of this study's results and the LT-GNN data were also performed in R.

Supplementary Tables

Table S1: Total Mortality by Tree Count from ADS

**YEAR**	ADS Reported Mortality [Million Trees]
2018	18
2017	27
2016	62
2015	27.6
2014	3.3
2013	1.53
2012	1.75
2011	1.66
2010	3.12
2009	1.40
2008	0.63
2007	9.09
2006	5.56

Data from https://www.fs.usda.gov/detail/r5/forest-grasslandhealth/?cid=fsbdev3_046696

Table S2: Upper bound SD biomass feedstock by HHZ in all counties

	Feasible [1000 BDT]		*Cost Effective [1000 BDT]*	
County	**HHZ Tier 1**	**HHZ Tier 2**	**HHZ Tier 1**	**HHZ Tier 2**
Tulare	1,072.4	9,640.2	464.1	2,607.5
Fresno	496.8	9,777.1	200.9	3,536.5
Madera	683.1	8,121.0	385.7	3,264.2
Tuolumne	1,219.7	6,012.3	563.1	2,453.7
Mariposa	842.5	3,706.7	432.1	1,592.1
Kern	327.1	2,013.3	144.9	709.7
Siskiyou	154.8	1,988.6	92.8	1,002.8
Calaveras	1,017.1	2,308.1	482.6	1,002.4
El Dorado	318.8	1,853.7	158.6	899.0
Modoc	175.9	1,882.9	152.4	1,489.4
Plumas	291.5	1,568.8	164.3	765.1
Lassen	86.1	1,136.0	72.9	984.0
Shasta	137.4	821.9	103.4	573.8
Placer	163.6	891.9	96.6	428.2
Trinity	158.2	749.6	51.9	206.8
Tehama	163.9	735.3	83.7	330.8
Humboldt	29.8	221.3	13.7	76.8
Amador	275.4	565.4	135.9	270.1
Sierra	69.4	532.4	28.5	185.3
Nevada	127.2	508.1	70.7	240.3
Butte	98.3	491.0	46.2	189.5
Del Norte	2.9	52.5	0.7	10.1
Mendocino	9.2	152.8	3.1	54.7
Mono	73.6	207.0	37.7	135.6
Glenn	5.1	119.1	2.8	50.2
Yuba	52.9	202.6	27.2	83.1
Sonoma	15.9	88.8	3.7	19.2
Ventura	7.7	176.9	4.8	88.2
Lake	40.3	131.7	26.1	78.9
Alpine	44.4	111.5	14.4	37.2
San Bernardino	29.0	69.9	16.6	35.8
San Luis Obispo	20.1	42.5	16.1	25.5
San Benito	7.4	80.1	3.5	31.7
Monterey	9.2	42.7	4.0	13.1
Santa Barbara	4.5	31.8	2.1	10.9
Los Angeles	7.7	37.9	3.0	10.5
Riverside	11.1	24.3	4.2	8.7
Colusa	1.0	38.6	0.5	19.2
San Diego	6.1	29.0	3.8	17.8
Inyo	0.3	19.5	0.2	7.6
Marin	0.7	0.0	0.1	-
Santa Clara	1.5	5.8	0.5	2.2
Alameda	3.3	7.6	1.0	3.0
Napa	1.3	2.6	0.7	1.6
Santa Cruz	1.4		0.4	-
San Mateo	0.0		0.0	-

Yolo		0.2	-	0.1
Contra Costa	0.1		0.1	-
San Joaquin	0.0		0.0	-

The column "Feasible" lists SD biomass after filtering for scattered pixels, wilderness/National Park status, VPT > 11.32 m^3, and dead tree density of < 2.5 dead trees per ha. The column "Cost Effective Total" lists feasibly harvestable SD biomass available at slopes < 40% and average tree volumes < 2.26 m^3. These values are all from the upper bound (UB) values, which uses the assumption that ADS detects all trees > 25 cm DBH with equal likelihood.

Table S3: SD biomass for all counties

County	Gross [1000-BDT]		Feasible [1000-BDT]		Cost-effective [1000-BDT]	
	DBH > 25 cm (UB)	DBH > 2.5 cm (LB)	DBH > 25 cm (UB)	DBH > 2.5 cm (LB)	DBH > 25 cm (UB)	DBH > 2.5 cm (LB)
Tulare	22,865.97	6,739.32	12,619.35	3,634.05	3,553.94	1,056.64
Fresno	15,137.39	4,700.47	11,410.03	3,557.83	3,941.96	1,293.08
Madera	10,134.32	3,191.55	8,595.20	2,713.83	3,460.22	1,081.50
Tuolumne	7,733.82	1,879.19	6,339.31	1,492.30	2,582.93	635.80
Mariposa	7,401.15	1,883.15	4,479.77	1,010.20	1,849.42	423.05
Kern	3,672.66	1,071.61	3,219.61	911.54	1,212.66	348.48
Siskiyou	3,482.85	895.16	2,578.79	638.54	1,219.69	313.49
Calaveras	2,468.00	534.67	2,365.04	509.03	1,028.68	231.78
El Dorado	2,391.92	601.44	2,229.64	551.76	1,067.90	273.28
Modoc	2,354.69	705.73	2,190.76	640.91	1,745.37	502.94
Plumas	2,089.01	485.11	1,808.52	409.45	888.26	211.09
Lassen	1,413.83	340.69	1,258.00	292.82	1,093.35	255.43
Shasta	1,477.03	322.21	1,056.86	221.04	720.81	154.56
Placer	1,102.86	262.59	959.07	217.31	455.13	111.59
Trinity	1,594.36	337.44	874.20	169.13	244.93	48.98
Tehama	1,067.60	243.20	874.16	193.02	381.19	85.38
Humboldt	1,158.92	247.33	788.25	152.87	362.96	70.69
Amador	869.67	194.01	741.12	154.24	343.70	74.94
Sierra	811.11	192.20	702.01	160.10	250.34	61.15
Nevada	663.05	148.96	555.52	113.32	260.96	56.89
Butte	584.78	98.04	524.70	84.74	205.12	35.93
Del Norte	422.17	81.41	282.28	43.01	87.84	14.77
Mendocino	436.15	107.49	281.57	61.84	99.27	23.78
Mono	456.28	118.23	245.75	68.14	157.35	46.60
Yuba	241.13	37.20	217.57	32.72	90.75	14.36
Glenn	249.81	59.21	219.16	48.64	89.15	20.62
Sonoma	277.49	51.68	215.69	35.94	46.60	8.09
Ventura	357.69	78.19	204.60	47.47	99.89	24.06
Lake	267.77	60.32	187.17	37.59	99.25	20.19
Alpine	448.46	133.03	172.46	47.16	54.95	15.70

County	Gross [1000-BDT]		Feasible [1000-BDT]		Cost-effective [1000-BDT]	
	DBH > 25 cm (UB)	DBH > 2.5 cm (LB)	DBH > 25 cm (UB)	DBH > 2.5 cm (LB)	DBH > 25 cm (UB)	DBH > 2.5 cm (LB)
San Bernardino	161.56	46.70	105.64	26.08	44.31	11.23
San Benito	132.35	50.28	100.40	36.06	41.51	15.03
San Luis Obispo	184.19	64.88	100.72	35.18	50.85	19.54
Monterey	180.77	45.86	83.52	22.45	25.22	8.29
Santa Barbara	117.83	36.79	68.11	19.32	20.93	7.44
Los Angeles	128.35	25.89	61.25	9.38	19.50	3.26
Riverside	90.02	24.55	42.09	9.85	14.61	3.59
Colusa	50.34	9.57	40.08	6.53	19.40	3.29
San Diego	114.86	42.89	38.34	9.39	23.16	6.16
Inyo	133.38	37.76	24.75	4.69	10.53	1.75
Marin	62.12	19.41	16.71	2.42	0.68	0.13
Santa Clara	27.55	9.95	11.22	3.34	3.95	1.25
Alameda	13.01	4.10	10.91	3.40	4.04	1.31
Napa	13.07	3.17	8.96	1.76	5.05	1.02
Santa Cruz	13.17	4.74	7.74	2.60	1.78	0.59
San Mateo	15.3	4.8	3.8	1.3	0.9	0.3
Yolo	5.9	1.9	2.6	1.0	1.2	0.7
Contra Costa	1.9	0.4	1.0	0.2	0.3	0.1
San Joaquin	0.1	< 0.1	0.1	< 0.1	< 0.1	< 0.1
Stanislaus	< 0.1	< 0.1	< 0.1	< 0.1	< 0.1	< 0.1
Orange	3.0	2.5	0.0	0.0	0.0	0.0
Solano	0.2	0.1	0.0	0.0	0.0	0.0
Sacramento	0.1	0.1	0.0	0.0	0.0	0.0
Merced	< 0.1	< 0.1	0.0	0.0	0.0	0.0
Kings	< 0.1	< 0.1	0.0	0.0	0.0	0.0

Table S4: SD biomass available in National Park Service (NPS) areas outside of wilderness designated areas

National Park	DBH > 25 cm (UB) [x10^6-BDT]	DBH > 2.5 cm (LB) [x10^6-BDT]
Sequoia	1,341.2	429.1
Yosemite	941.0	210.1
King Canyon	211.4	63.6
Lassen Volcanic	76.8	18.7
Redwood	76.6	16.3
Whiskeytown-Shasta-Trinity	57.0	10.1
Point Reyes	9.5	3.7
Golden Gate	6.9	2.2
Pinnacles	0.5	0.1
Lava Beds	0.1	0.1
Devils Postpile	0.1	< 0.1
Muir Woods	< 0.1	< 0.1
Death Valley National Park	< 0.1	< 0.1

Table S5: SD biomass in federally designated wilderness areas

Wilderness area	DBH > 25 cm (UB) [x10^6-BDT]	DBH > 2.5 cm (LB) [x10^6-BDT]
Sequoia-Kings Canyon	4282.2	1298.3
Yosemite	3331.9	1026.7
Ansel Adams	1824.3	539.5
John Krebs	1357.6	402.2
Golden Trout	1229.5	405.1
John Muir	1106.6	336.7
Trinity Alps	712.2	160.5
Kaiser	504.9	148.6
Marble Mountain	332.2	93.4
Carson-Iceberg	234.2	70.7
Yolla Bolly-Middle Eel	229.7	59.4
Lassen Volcanic	216.6	45.9
Domeland	210.9	78.9
Monarch	200.8	54.7
Siskiyou	157.2	42.3
Mokelumme	144.9	44.9
Owens Peak	116.1	25.9
Jennie Lakes	114.1	24.1
South Warner	109.3	42.2
Owens River Headwaters	102.2	23.1
Sespe	90.7	13.3
Chimney Peak	87.6	26.5
Kiavah	82.2	30.8
South Sierra	65.0	25.3
Emigrant	64.8	18.6
Bucks Lake	63.5	16.3
Chumash	59.2	15.2
Dinkey Lakes	55.8	16.1
Sacatar Trail	54.6	17.0
Thousand Lakes	51.9	12.5
Ventana	49.3	5.9
Caribou	39.7	8.9
Hoover	38.2	8.5
San Jacinto	36.7	11.2
Snow Mountain	31.9	8.5
Mt. Shasta	27.6	10.8
Phillip Burton	27.6	10.5
Inyo Mountains	26.3	9.8
Desolation	21.6	5.7
Yuki	20.9	4.1
Pleasant View Ridge	20.7	4.0
Granite Chief	20.2	5.0
Garcia	18.0	5.8
Russian	15.7	3.9
San Rafael	15.2	3.0
Ishi	14.5	2.0
Sheep Mountain	13.9	2.7
Cucamonga	12.4	3.5
Silver Peak	11.3	3.4

**Wilderness area**	**DBH > 25 cm (UB) [x10^6-BDT]**	**DBH > 2.5 cm (LB) [x10^6-BDT]**
Santa Lucia	8.1	2.5
San Gabriel	7.0	1.0
Chanchelulla	6.4	1.5
Machesna Mountain	6.3	1.9
Dick Smith	6.1	1.3
King Range	5.0	1.0
San Gorgonio	4.4	1.2
Cache Creek	2.1	0.2
North Fork	1.8	0.3
South Fork San Jacinto	1.3	0.4
Bighorn Mountain	1.1	0.3
Matilija	0.7	0.1
White Mountains	0.7	0.5
Bright Star	0.6	0.1
Pine Creek	0.6	0.2
Hain	0.5	0.1
Sanhedrin	0.5	0.3
Elkhorn Ridge	0.5	0.1
Cedar Roughs	0.2	< 0.1
Agua Tibia	0.1	0.1
Magic Mountain	0.1	< 0.1
Mount Lassic	0.1	< 0.1
Lava Beds	< 0.1	< 0.1
Cahuilla Mountain	< 0.1	< 0.1
South Fork Eel River	< 0.1	< 0.1
San Mateo Canyon	< 0.1	< 0.1
Santa Rosa	< 0.1	< 0.1
Hauser	< 0.1	< 0.1
Sawtooth Mountains	< 0.1	< 0.1
Piper Mountain	< 0.1	< 0.1

Table S6: Dead and live biomass in the ADS surveyed areas organized by method of estimation

Total	CRM, DBH > 25 cm (UB) [x10^6-BDT]	CRM, DBH > 2.5 cm (LB) [x10^6-BDT]	Jenkins, DBH > 25 cm (UB) [x10^6-BDT]	Jenkins, DBH > 2.5 cm (LB) [x10^6-BDT]
Live	470.0	533.6	620.1	720.0
Dead	95.1	26.2	100.0	26.3
Percent dead	20.2%	4.9%	16.1%	3.7%

Table S7: California energy mix of 2016

Fuel Type	California In-State Generation	California In-State Generation (%)	Northwest Import	Southwest Imports	California Energy Mix	California Power Mix
Coal	324	0.16%	373	11,310	12,006	4.13%
Large Hydro	24,410	12.31%	3,367	1,904	29,681	10.21%
Natural Gas	98,831	49.86%	41	7,120	105,992	36.48%
Nuclear	18,931	9.55%	0	7,739	26,670	9.18%
Oil	37	0.02%	0	0	37	0.01%
Other	394	0.20%	0	0	394	0.14%
Renewables	55,300	27.90%	11,710	6,952	73,961	25.45%
Biomass	5,868	2.96%	659	25	6,553	2.26%
Geothermal	11,582	5.84%	96	1,038	12,717	4.38%
Small Hydro	4,567	2.30%	229	1	4,796	1.65%
Solar	19,783	9.98%	0	3,791	23,574	8.11%
Wind	13,500	6.81%	10,725	2,097	26,321	9.06%
Unspecified	N/A	N/A	26,888	14,937	41,825	14.39%
Total	198,227	100.00%	42,378	49,963	290,567	100.00%

All values in GWh, unless otherwise specified. Table contents were obtained from http://www.energy.ca.gov/almanac/electricity_data/total_system_power.html.

Supplementary Figures

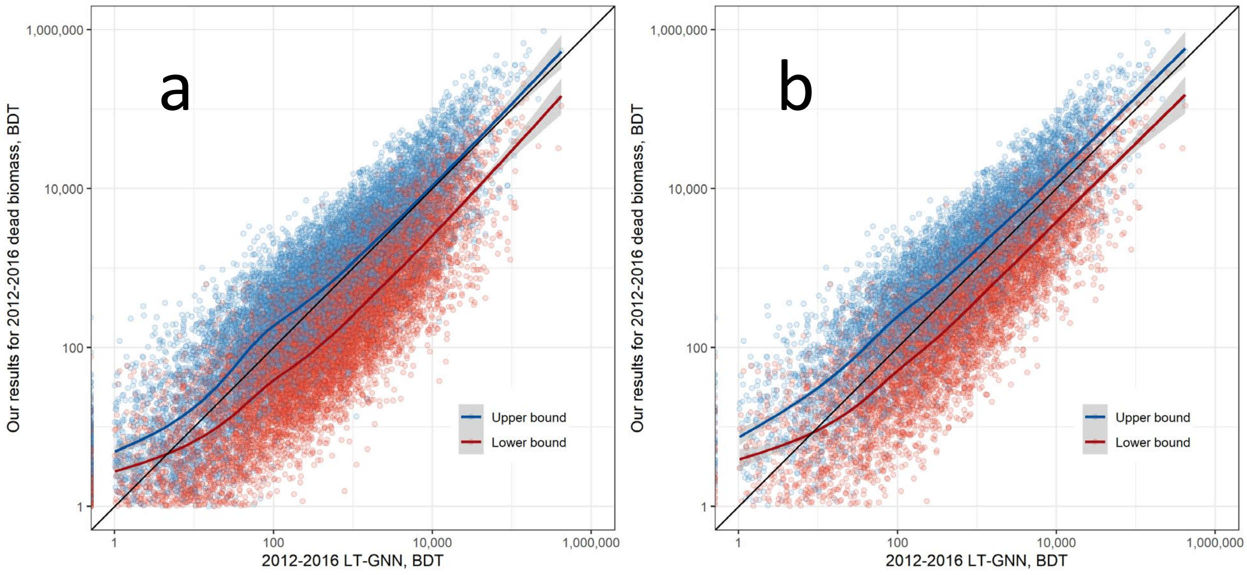

Figure S1. Comparison of our results to LT-GNN results for a) all counties and b) Priority Counties. Though we compared biomass loss across 2012-2016, we only examined areas within 2016 ADS polygons because each year's ADS polygons often overlap with each other, which would result in excessive pseudo-replication of the validation. The black line represents a 1:1 fit, while the blue and red lines represent generalized additive model fits for the upper and lower bounds, respectively, which were modeled using cubic splines according to the function "geom_smooth" in the R package "ggplot2." Gray shading represents 95% confidence intervals.

References

1. U.S. Forest Service. U.S. Forest Service Pacific Southwest Region Forest Health Protection Aerial Detection Survey. (2017).
2. US Forest Service. *2016 Aerial Survey Results: California*. (United States Department of Agriculture, 2017).
3. US Forest Service. *Aerial Survey Geographic Information System Handbook*. (United States Department of Agriculture, 2005).
4. McConnell, T. J., Johnson, E. W. & Burns, B. Guide to Conducting Aerial Sketchmapping Surveys. (2000).
5. Landscape Ecology, Modeling, Mapping and Analysis (LEMMA) Group & U.S. Forest Service. Wilderness & Wild and Scenic Rivers & Wilderness Study Areas. (20142017).
6. Bechtold, W. A., Patterson, P. L. The enhanced forest inventory and analysis program - national sampling design and estimation procedures. *Gen Tech Rep SRS-80 Asheville NC US Dep. Agric. For. Serv. South. Res. Stn. 85 P* **080**, (2005).
7. Ohmann, J. L. *et al.* Mapping change of older forest with nearest-neighbor imputation and Landsat time-series. *For. Ecol. Manag.* **272**, 13–25 (2012).
8. Ohmann, J. L., Gregory, M. J. & Roberts, H. M. Scale considerations for integrating forest inventory plot data and satellite image data for regional forest mapping. *Remote Sens. Environ.* **151**, 3–15 (2014).
9. Woodall, C. W., Heath, L. S., Domke, G. M. & Nichols, M. C. *Methods and equations for estimating aboveground volume, biomass, and carbon for trees in the US forest inventory, 2010*. (U.S. FOREST SERVICE, 2011).
10. Ester, M., Kriegel, H.-P., Sander, J., Xu, X. & others. A density-based algorithm for discovering clusters in large spatial databases with noise. in *Kdd* **96**, 226–231 (1996).
